# Use of Liquid Chromatography with Electrochemical Detection for the Determination of Antioxidants in Less Common Fruits

**DOI:** 10.3390/molecules131102823

**Published:** 2008-11-14

**Authors:** Zbynek Gazdik, Vojtech Reznicek, Vojtech Adam, Ondrej Zitka, Tunde Jurikova, Boris Krska, Jan Matuskovic, Jan Plsek, Jan Saloun, Ales Horna, Rene Kizek

**Affiliations:** 1 Department of Breeding and Propagation of Horticultural Plants, Faculty of Horticulture, Mendel University of Agriculture and Forestry, Valtická 337, CZ-691 44 Lednice, Czech Republic; E-mails: xgazdik@node.mendelu.cz (Z. C.), reznicek@node.mendelu.cz (V. R.); 2 Department of Chemistry and Biochemistry, Faculty of Agronomy, Mendel University of Agriculture and Forestry, Zemedelska 1, CZ-613 00 Brno, Czech Republic; E-mail: ilabo@seznam.cz (V. A.); 3 Department of Agrochemistry, Soil Science, Microbiology and Plant Nutrition, , Faculty of Agronomy, Mendel University of Agriculture and Forestry, Zemedelska 1, CZ-613 00 Brno, Czech Republic; 4 Department of Animal Nutrition and Forage Production, Faculty of Agronomy, Mendel University of Agriculture and Forestry, Zemedelska 1, CZ-613 00 Brno, Czech Republic; 5 Institute of Natural and Informatics’ Sciences, Faculty of Central European Studies, Constantine the Philosopher University in Nitra, Nabrezie mladeze 91, SK-949 76 Nitra, Slovak Republic; E-mail:; 6 Department of Fruit Growing, Faculty of Horticulture, Mendel University of Agriculture and Forestry, Valtická 337, CZ-691 44 Lednice, Czech Republic,; 7 Department of Fruit Production, Viticulture, and Enology, Horticulture and Landscape Engineering Faculty, University of Agriculture in Nitra, Trieda A. Hlinku 2, SK-949 76 Nitra, Slovak Republic; 8 Department of Applied Pharmacy, Faculty of Pharmacy, University of Veterinary and Pharmaceutical Sciences, Palackeho 1 - 3, CZ-612 42 Brno, Czech Republic; 9 Tomas Bata University, T.G. Masaryka 275, CZ-762 72 Zlin, Czech Republic

**Keywords:** Phenolic compound, Electrochemical detection, Less common fruit species, liquid Chromatography, Neuroprotective food

## Abstract

Neurodegenerative disorders (NDD) have become the common global health burden over the last several decades. According to World Health Organization (WHO), a staggering 30 million people will be affected by Alzheimer’s disease in Europe and the USA by 2050. Effective therapies in this complex field considering the multitude of symptoms associated with NDD indications, have not been found yet. Based on the results of NDD related studies, prevention appears to be the promise alternative. Antioxidative and anti-inflammatory properties are hypothesized for natural phenolics, a group of plant secondary products that may positively impact neurodegenerative diseases. In these studies, phenolic-rich extracts from less common fruit species: Blue honeysuckle (*Lonicera edulis*, Turcz. ex. Freyn), Saskatoon berry (*Amelanchier alnifolia* Nutt.), and Chinese hawthorn (*Crateagus pinnatifida* Bunge) were obtained and analyzed to detect neuroprotective substances content and establish a potential therapeutic value. High performance liquid chromatography with electrochemical detection was optimized and further applied on analysis of the extracts of less common fruit species. It was observed that Chinese hawthorn and Blue honeysuckle extracts are potent source of neuroprotective phenolic antioxidants. In accordance the results, it appears that the fruit or formulated products may have the potential for the prevention of neurodegenerative diseases.

## Introduction

Recently, much interest has been generated in drug discovery and development from progress in the global propagation of neurodegenerative disorders (NDD). The number of people with neurological sequelae of nutritional disorders and neuropathies (352 million) and neurological sequelae secondary to injuries (170 million) also add substantially to the above burden. This trend, recorded over the several past decades [1], have led to acceleration in the search for therapeutic treatments, because none of the conventional strategies, nor any new pharmaceuticals (HST, CEI) have changed the complex problem with growing numbers of NDD-medicated patients.

The nervous system, including the brain, spinal cord, and peripheral nerves, is rich in both unsaturated fats (which are prone to oxidation) and iron [2]. The high lipid content of nervous tissue, coupled with its high metabolic (aerobic) activity, makes it particularly susceptible to oxidative damage [3]. The high level of brain iron can lead to oxidative stress via the iron-catalyzed reduce hydrogen peroxide to the highly reactive hydroxyl radical [4,5]. It has been shown that reactive oxygen species (ROS) are the important mediators of cell signalling events such as inflammatory reactions (superoxides) and the maintenance of vascular tone (nitric oxide) [6,7,8]. Excessive production of ROS, outstripping endogenous antioxidant defence mechanisms, is referred to as oxidative stress [9]. Overproduction of ROS has been associated with the pathogenesis of variety of neurodegenerative disorders such as cognitive failures in Alzheimer’s disease (AD) [10,11,12,13,14,15], retinal degeneration, ischemic dementia [6,7,9], Parkinson’s disease (PD) [16], and amyotrophic lateral sclerosis (ALS, "Lou Gehrig's disease") [17]. Although significant relationships between oxidative damage and neurodegenerative processes have been identified or are suspected, important pathways of relationship between β-amyloid production and exposure of hippocampal neurones to ROS attack has not been clarified yet [18,19,20,21], Reduction of oxidative stress and inflammation show the way of new therapeutic strategy for inhibiting the pathogenetical cascade associated with a multitude of symptoms such as brain atrophy, cerebrovascular hemodynamics, cognitive decline, inflammation-induced apoptosis, lipid metabolism dyshomeostasis, and amyloidal deposition and others. The situation has shown the necessity of precaution of the diseases. The importance of neuroprotective diet has been accepted by the public recently. Rather, current therapeutic strategies under investigation for AD include inhibitors of Ap production, compounds that prevent its oligomerization and fibrillization, anti-inflammatory drugs, inhibitors of cholesterol synthesis, antioxidants, neurorestorative factors and vaccines [22,23].

Free radicals are reactive organic or inorganic molecules with one or more unpaired electrons, commonly formed in the body as a result of metabolic processes which are normally eliminated by the antioxidant line of the body’s defence systems [24]. Free radical scavengers are compounds that are capable of donating electrons or hydrogen atoms to inhibit a free radical reaction [2]. A number of *in vitro* models [25,26], followed by *in vivo* studies [27,28,29], have shown that antioxidants, both endogenous and dietary, can protect neurocerebral system against oxidative stress [30,31]. In addition, the research suggests that the phenolic compounds found mainly in fruits such as berries, may exert protection against NDD-related deficits in cognitive and motor function [20,22,23,26].

Considerable epidemiological evidence suggests that regular consumption of fruit and vegetables decreases the risk of developing several neurodegenerative disorders. It has also been shown that the antioxidant activity varies with the types of phenolic compounds present in the fruit. Certain types of phenolic compounds show greater antioxidant activity than others [32,33]. Less common fruit species or alternative fruit crops are characterized by resistance to both abiotic and biotic factors of an environment and valuable biochemical composition of fruits [34,35,36,37,38]. The lack of data in food composition leads to difficulties in quantifying the daily polyphenol intake. Methods to quantify the total polyphenol content of plant food products have been recently proposed [39,40,41,42,43,44,45,46,47,48,49,50,51,52,53]. It is extremely important to develop effective method to obtain novel opportunities for NDD prevention through the screening of every single target from a complex field of chemical diversity of plant substances. High performance liquid chromatographic analysis are most commonly employed to identify antioxidants in beverage samples using UV detection and to quantify them [54,55,56,57]. Significantly lower detection limits have been achieved by electrochemical detection [40,58,59,60,61].

**Figure 1:**
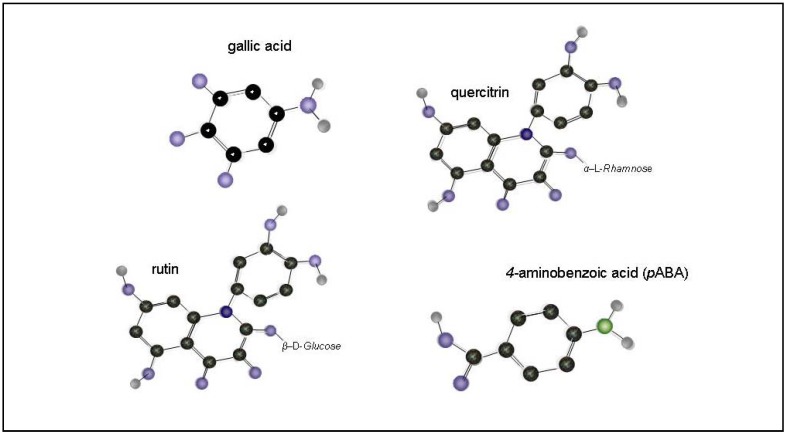
Structure of rutin, quercitrin, gallic acid and 4*-*aminobenzoic acid.

Gallic acid (3,4,5-trihydroxybenzoic acid, (GA, Figure 1) is a naturally occurring antioxidant that has shown a multitude of biological activities [62,63], including neuroprotective ones. Analysis of cell apoptosis, intracellular GSH levels, production of ROS and the influx of Ca^2+^ under oxidative stress had confirmed protective effects of gallic acid and it’s derivatives in cell systems [64]. Over the several past decades, *p*-aminobenzoic acid (*p*ABA, Figure 1) has been frequently discussed as a compound that is an essential nutrient for microorganisms and some animals, but has not been shown to be essential for people. Recently (*p*ABA) has been well established a potent neutralizer of singlet molecular oxygen, a potent free radical which is a common by-product of normal metabolism [65,66,67,68]. More than 4,000 different polycyclic hydroxyphenols have been described to occur in food of plant origin [69]. Quercitrin (quercetin-3-*O*-a-L-rhamnoside, Figure 1) is the glycoside form derived from the pentahydroxyflavone quercetin that has been extensively studied over the past 30 years [70]. Quercetin is the flavonol that seems to be the most powerful flavonoid for protecting the body against reactive oxygen species [17,71,72]. Quercetin (Figure 1), through its catechol-*O*-methyltransferase (COMT) and monoamine oxidase (MAO) enzymes inhibiting properties, might potentiate the anticatabolic effect of L-dopa plus carbidopa treatment. Thus, quercetin can serve as an effective adjunct to L-dopa therapy in Parkinson disease [73]. Because plants contain many different classes and types of antioxidants, knowledge of total content of components to scavenge free radicals, would be useful for epidemiologic purpose [21,23,74,75,76,77]. In this regard, there is an urgent need to improve analytical methods to quantify various food flavonoids and their metabolites in biological fluids [78]. Liquid chromatography or electrophoresis with various types of detectors [40,41,42,59,79], or stationary electrochemical methods [40,58,60,61] are used for their determination.

**Figure 2:**
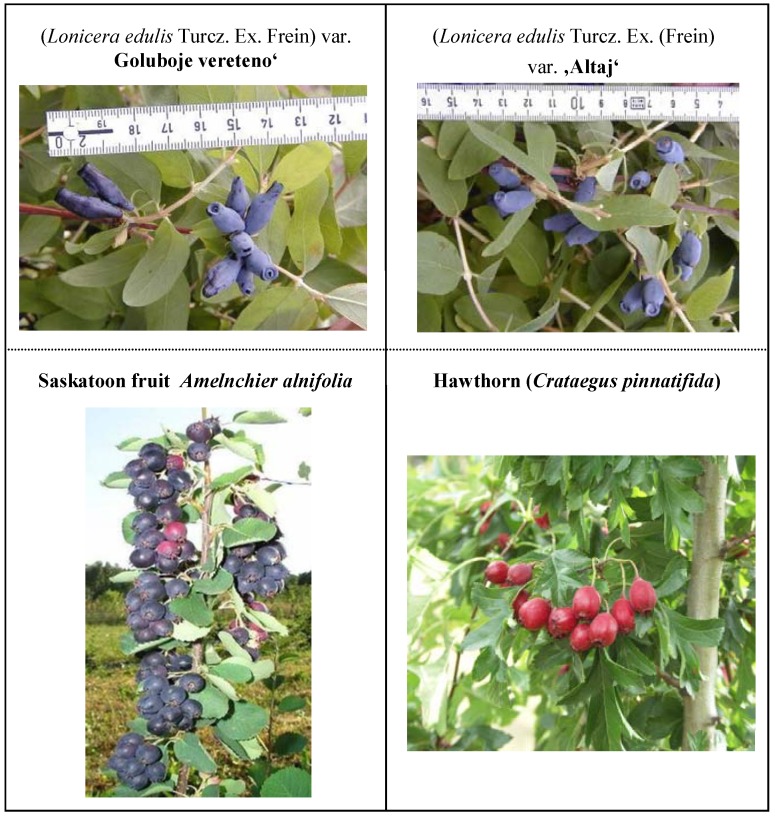
Analyzed fruit samples.

This study was focused on the determination of neuroprotective plant phenols (rutin, quercitrin, gallic acid and 4*-*aminobenzoic acid, Figure 1) in fruits of three less examined plant species: Blue honeysuckle (*Lonicera edulis*, Turcz. ex. Freyn), Saskatoon berry (*Amelanchier alnifolia* Nutt.) and Chinese hawthorn (*Crateagus pinnatifida* Bunge) (Figure 2). The target analytes have been reported to possess a multitude of biological activities, including antiallergic, anti-inflammatory, antiviral, antiproliferative, and anticarcinogenic activities with a beneficial effects on mammalian metabolism [80,81,82], the antioxidant and anti-inflammatory activities in neurocerebral system contributes to their bioavailability and the dosage [78]. Keeping out the sequence of conditions involving the intestinal transport as well as structurally-specific mechanisms of target’s absorption [83] and their mobility through the bloodstream, the ability to penetrate the hematoencephalic barrier plays the key role for neuroprotective effect [63,64]. Gallic acid, some *p*ABA derivates, rutin and quercitrin meet these requirements.

(A) (*Lonicera edulis* Turcz. Ex. Frein) var. “Goluboje vereteno“. (B) (*Lonicera edulis* Turcz. Ex. Frein) var. “Altaj”. (C) Saskatoon berry (*Amelanchier alnifolia* Nutt). (D) Hawthorn (*Crataegus pinnatifida*).

## Results and Discussion

Antioxidants are known to take part in reducing reactions. Substances without the electrochemical activity are antioxidant activity less, on the other hand, the activity is expected from the sort of substances disposing low levels in half-undulate signal. Because there exists significant relation in between the substance antioxidant activity and the ability to provide electrochemical signal [32,78,84,85], the selective and sensitive electrochemical detection coupled with high performance liquid chromatography (HPLC-ED) presents an optimal analytical tool for their detection.

### Identification and quantification of phenolic compounds in fruits by using HPLC-ED

**Figure 3:**
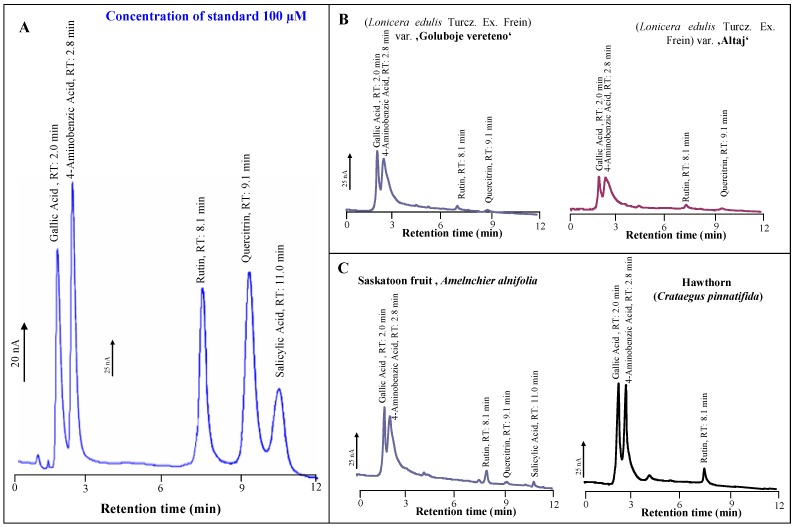
(A) Typical HPLC-ED chromatogram of rutin, quercitrin, gallic acid and 4*-*aminobenzoic acid, and salicylic acid. (B, C) Typical HPLC-ED chromatograms of plant extracts.

Primarily we focused on the study of basic electrochemical behaviour of compounds of interest (rutin, quercitrin, gallic acid and *4-*aminobenzoic acid – *p*ABA). Through the hydrodynamic voltammogram we characterized the oxidative signals and optimized frequency a pH of mobile phase for sensitive determination of phenolic antioxidants. We applied potentials from 100 mV to 950 mV (per 50 mV) on working glassy carbon electrode. We observed oxidative signals associated with electrochemical conversion of hydroxyl groups of the target molecules [40,86]. The signals appeared already at 200 mV. To get sufficient sensitivity we choose 950 mV for the following experiments (Figure 3A).

### Content of phenolic compounds in less common fruits

Separation of target molecules was carried out on a Restek Allure C18 column with reverse-phase elution. The solution of methanol - 60 % and (0.065 M) acetic acid - 40 % (*v*/*v*) was used as mobile phase. The conditions are given: column and detector temperature 55°C, flow rate of the mobile phase 1 mL/min, working electrode potential 950 mV. Under these conditions we analysed extracts from *Crataegus pinnatifida, Lonicera edulis and Amelanchier alnifolia* (Figure 3B, C). In the extract of Chinese Hawthorn (*Crataegus Sp*.) we determined that content of gallic acid was almost three times higher, compared to those of *p*ABA and rutin (Table 1), whereas quercitrin was not detected. *In vivo* quercitrin can be more important antioxidant and neuroprotective agent than quercetin because of its high bioavailability in the digestive tract [26].

**Table 1:** Levels of neuroprotective antioxidants*.

Fruit	Compound	Level of phenols (mg/kg)
(*Crataegus pinnatifida* BUNGE)	gallic acid	2,640
	*4-*aminobenzoic acid	970
	rutin	910
	quercitrin	n.d.
(*Lonicera edulis,* Turcz. ex. Freyn)	gallic acid	600
	*4-*aminobenzoic acid	170
	rutin	240
	quercitrin	220
(*Amelanchier canadensis* L.)	gallic acid	240
	*4-*aminobenzoic acid	20
	rutin	230
	quercitrin	230

*Twenty samples obtained from each fruit were analysed in triplicate; n.d.: not detected

A typical HPLC-ED chromatogram of the extract from *Crataegus pinnatifida* is shown in Figure 3C. Higher content of quercitrin was detected in *Lonicera edulis* and *Amelanchier alnifolia* fruits in tens of mg per 100 g of fresh weight. Quercitrin has been found in a wide range of berries such as whortleberry (158 mg/kg fresh weight), lingonberry (74 and 146 mg/kg), cranberry (83 and 121 mg/kg), chokeberry (89 mg/kg), sweet rowan (85 mg/kg), rowanberry (63 mg/kg), sea buckthorn berry (62 mg/kg) and crowberry (53 and 56 mg/kg) [54]. In addition, the highest content in *Lonicera edulis* and *Amelanchier alnifolia* was of gallic acid (Table 1). According to Pokorna and Matuskovic the level of the aglycone rutin obtained at several genotypes of *Lonicera* Sp. does not surpass a level of 300 mg/kg [87]. *para*-Aminobenzoic acid helps the body generate important nutrients such as vitamin K, folic acid and thiamine through stimulation the intestinal bacteria, enabling them to produce folic acid, assists in the formation of red blood cells which carry oxygen to sensitive brain tissue and to all parts of the body. The typical therapeutic dosage of *p*ABA is 300 to 400 mg daily [88]. From the point of view of total phenolic content, the fruits of *Crataegus pinnatifida* had an almost four times higher content of neuroprotective phenolics, compared to the other plant species we investigated. This can account for the considerable antioxidant activity of this plant species.

## Experimental

### Chemicals

Flavonoid standards for HPLC: gallic acid and 4-aminobenzoic acid (Sigma Aldrich Corp., USA), rutin trihydrate and quercitrin dihydrate (Roth GmbH, Karlstruhe, Germany) were used. Methanol (>99.9%; *v*/*v*), acetic acid, formic acid for HPLC and all other chemicals used were purchased from Sigma Aldrich.

### High performance liquid chromatography with electrochemical detection (HPLC-ED)

The instrument used consisted of a solvent delivery pump (ESA Inc., Model 582, Chelmsford, MA, USA), a guard cell (ESA Inc., Model 5020), a Metachem Restek Allure reverse-phase chromatographic column (150.0 × 4.6 mm, 5 μm particle size, Canada), and an electrochemical detector. The electrochemical detector includes one low volume flow-through analytical cells (ESA Inc., Model 5040), which is consisted of glassy carbon working electrode, hydrogen-palladium electrode as reference electrode and auxiliary carbon electrode, and Coulochem III as a control module. The samples (5 μL) were injected using an autosampler (ESA Inc., Model 540 Microtiter HPLC). The data obtained were treated by CSW 32 software (Version 1.2.4, Data Apex, Czech Republic). Guard cell potential was set as 0 V. The glassy carbon electrode was polished mechanically with 0.1 μm alumina (ESA Inc.) and sonicated at room temperature for 5 min using a Sonorex Digital 10 P Sonicator (Bandelin, Berlin, Germany) at 40 W.

### Biological material

The following fruit samples was analysed for a neuroprotective content evaluation: i) one genotype of Blue Honeysuckle (*Lonicera edulis*, Turcz. Ex. Freyn). The fruit was harvested in mid-May and stored at -18 ºC for seven months. ii) One variety of Saskatoon berry (*Amelanchier alnifolia* Nutt.). The fruit was harvested during July; after the harvest the fruit was stored at -18 ºC, for five months. iii) One variety of (*Crateagus pinnatifida* Bunge). The fruit was harvested in early October and stored at -18 ºC, for three months.

(*Lonicera edulis* Turcz. Ex. Frein) var. “Goluboje vereteno“. This variety as a seedling obtained from free pollination has its origin in the “Sady Sibiri” orchards. The roundish weak branched shrubs are round shaped and shoot growth is fast. The level of frost resistance is high. The long berries with soft pulp are sweet, sour and lightly bitter. The yield is 2.5 kg.

(*Lonicera edulis* Turcz. Ex. Frein) var. “Altaj” is a hybrid variety obtained from breeding of *Lonicera kamtschatica* × *Lonicera turczaninowii.* This compact shrub exhibits dynamical growth and a high level of frost resistance. Hedge berries are cylindrically shaped, dark blue, with 1.05 g mean weight values. The pulp is juicy, sweet and sour, with an intense aroma. The harvest period comes very early; the shrub provides yield about 2.8 kg.

Saskatoon berry (*Amelnchier alnifolia*). *Amelanchier alnifolia* Nutt. is a tree or shrub providing good yields of purple, dark blue or black fruit so-called also Juneberries. There can be a multitude of levels in the fruit size, ranging on average from 0.6-1.6 mm. The thin-peeled fruit is very sweet and juicy, similar to commonly grown blueberries. Generally, *Amelanchier* fruit is known to be used by healers in folk medicine.

Hawthorn (*Crataegus pinnatifida*). Chinese Hawthorn belongs to *Rosacea* family, that suggests a high content of phenolic plants constituents. Fruit size is about 40 mm, drupes are red with 4-5 seeds in the yellow pulp. *Crataegus* fruit use to be very popular in folk medicine and it is frequently used nowadays by healers in eastern parts of Asia.

### Sample preparation

The fruits of the analysed species were thawed at room temperature. Weighed fruits (approximately 6 g) were transferred to a mortar, and liquid nitrogen was added. The frozen samples were ground for 5 min. Then, 80% methanol (10 mL) was added to the mortar, and the sample was ground for 10 min. The homogenate was transferred to a test-tube and homogenised by shaking on a Vortex–2 Genie (Scientific Industries, New York, USA) at 4 °C for 30 min. The homogenate was centrifuged (14,000 g) for 30 min at 4 °C using a Universal 32 R centrifuge (Hettich-Zentrifugen GmbH, Tuttlingen, Germany). Before the analysis the supernatant was filtered through a membrane filter (0.45 μm Nylon filter disk, Millipore, Billerica, Mass., USA). Filtrate (150 µL) was diluted 1:400-1,080 with 80% methanol.

### Descriptive statistics

Data were processed using Microsoft Excel® (USA). Results are expressed as mean ± standard deviation (S.D.), unless stated otherwise.
